# Are serum cytokines early predictors for the outcome of burn patients with inhalation injuries who do not survive?

**DOI:** 10.1186/cc6932

**Published:** 2008-06-18

**Authors:** Gerd G Gauglitz, Celeste C Finnerty, David N Herndon, Ronald P Mlcak, Marc G Jeschke

**Affiliations:** 1 Shriners Hospitals for Children, 815 Market Street, Galveston, Texas, 77550, USA; 2 Department of Surgery, University of Texas Medical Branch, 301 University Boulevard, Galveston, Texas, 77550, USA

## Abstract

**Introduction:**

Severely burned patients suffering from inhalation injury have a significantly increased risk for mortality compared with burned patients without inhalation injury. Severe burn is associated with a distinct serum cytokine profile and alterations in cytokines that contribute to morbidity and mortality. The aim of the present study was therefore to determine whether severely burned pediatric patients with concomitant inhalation injury who had a fatal outcome exhibited a different serum cytokine profile compared with burn patients with inhalation injury who survived. Early identification followed by appropriate management of these high-risk patients may lead to improved clinical outcome.

**Methods:**

Thirteen severely burned children with inhalation injury who did not survive and 15 severely burned pediatric patients with inhalation injury who survived were enrolled in the study. Blood was collected within 24 hours of admission and 5 to 7 days later. Cytokine levels were profiled using multiplex antibody coated beads. Inhalation injury was diagnosed by bronchoscopy during the initial surgery. The number of days on the ventilator, peak inspiratory pressure rates, arterial oxygen tension (PaO_2_)/fraction of inspired oxygen (FiO_2_) ratio and incidence of acute respiratory distress syndrome were recorded for those patients.

**Results:**

Significantly altered levels of IL-4, IL-6, IL-7, IL-10, and IL-13 were detected within the first 7 days after admission in serum from burn pediatric patients with concomitant inhalation injury who did not survive when compared with similar patients who did (*P *< 0.05). Alterations in these cytokines were associated with increased incidence of acute respiratory distress syndrome, number of days under ventilation, increased peak inspiratory pressure, and lower PaO_2_/FiO_2 _ratio in this patient population. Multiple logistic regression analysis revealed that patients with increased IL-6 and IL-10 as well as decreased IL-7 serum levels had a significantly greater risk for mortality (*P *< 0.05).

**Conclusion:**

Early alterations in serum levels of IL-6, IL-7 and IL-10 may constitute useful predictive markers for identifying patients those who have sustained a burn with concomitant inhalation injury and who have high mortality.

## Introduction

Mortality from major burns has significantly decreased during the past 20 years. Inhalation injury, however, still constitutes one of the most critical adverse factors after thermal insult and has remained associated with a mortality rate of 25% to 50% when patients require ventilator support for more than 1 week after injury [1-3]. Although many organ systems are affected by a burn, the pulmonary system often sustains the most damage [4]. Because inhalation injury is a major contributor to mortality in thermally injured patients [3-5], early diagnosis and treatment are crucial for the prevention of complications. The arterial oxygen tension (PaO_2_)/fraction of inspired oxygen (FiO_2_) ratio is a parameter that is widely used to define acute respiratory distress syndrome (ARDS) and – along with age, underlying disease, malnutrition, and infection – it has been proposed to be a prospective clinical predictor of poor outcome after inhalation injury [6].

Inhalation injury is caused by steam or toxic inhalants such as fumes, gases, or mists. It results in increased pulmonary microvascular hyperpermeability, leading to edema formation, atelectasis, and tracheobronchitis [1,7]. Subsequently, neutrophils undergo diapedesis from the pulmonary microvasculature and release enzymes (including elastase) and free oxygen radicals, disrupting endothelial junctions and epithelial integrity, thus permitting an exudate of protein-rich plasma to enter the lungs [8]. The inhalation of toxic smoke leads to the release of inflammatory mediators such as thromboxane, which enhance pulmonary artery pressure and cause secondary damage to the respiratory epithelium and the release of additional mediators, such as tumor necrosis factor (TNF) [3,8]. Release of these inflammatory molecules into the systemic vasculature may cause injury to other organs [9].

In a previous study [10] we demonstrated that a burn causes marked alterations in various inflammatory cytokines 1 week after thermal injury when compared with healthy children. Alterations in inflammatory mediators, such as cytokines, are main contributors to the incidence of multiple organ failure and mortality in critically ill patients [11]. The aim of the present study was therefore to assess, in a cohort of severely burned pediatric patients with inhalation injury, whether those severely burned children who had a fatal outcome exhibited a distinct serum cytokine profile in comparison with those who survived; such a profile could serve as a predictive marker.

## Materials and methods

Thirteen severely burned children with inhalation injury who did not survive burn trauma ('nonsurvivors') and 15 severely burned children with inhalation injury who survived ('survivors') were enrolled in the study. Permission for conducting the study was obtained from the Institutional Review Board of the University of Texas Medical Branch, Galveston, Texas, USA. Before the study, for each participant the patient, parent, or child's legal guardian signed a written informed consent form. All patients were 16 years of age or younger and were admitted within 7 days after injury to the Shriners Hospital for Children, Galveston, Texas, USA. Every child was suffering from burns to more than 40% of total body surface area with a third degree component of more than 24%, and required at least one surgical intervention for escharectomy and skin grafting. Patients were excluded if there was any sign of infection, sepsis, concomitant major injuries, or complications at admission.

After admission, patients were treated according to the standard of burn care at our hospital, including early excision and grafting of the burn wound, and fluid and caloric resuscitation in accordance with the Galveston formulas [12].

### Demographics

Age, burn size, depth of burn, and time to admission were recorded in each group.

### Inhalation injury

**Figure 1:**
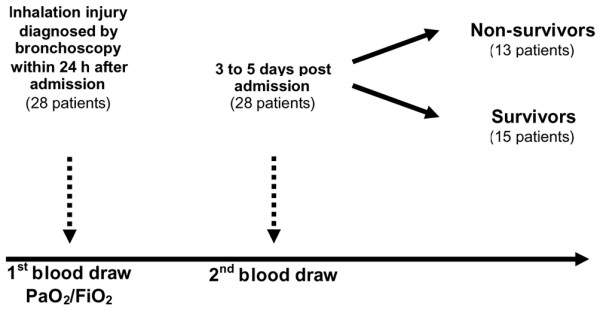
Outline of the study. The arterial oxygen tension (PaO_2_)/fraction of inspired oxygen (FiO_2_) ratio was measured in all patients within 24 hours after admission. Blood was drawn at hospital admission and 5 to 7 days afterward.

Inhalation injury was diagnosed by bronchoscopy, which was performed in all patients within 24 hours after admission in accordance with the following criteria (Figure 1): signs of exposure to smoke in an enclosed space, including presence of facial burns, singed nasal vibrissae, bronchorrhea, sooty sputum, and wheezing or rales upon auscultation; hypoxemia and/or elevated levels of carbon monoxide; and bronchoscopy findings of airway edema, inflammation, mucosal necrosis, presence of soot and charring in the airway, tissue sloughing, or carbonaceous material in the airway.

### PaO_2_/FiO_2 _ratio

The PaO_2_/FiO_2 _ratio was used to quantify the degree of abnormalities in pulmonary gas exchange. PaO_2_/FiO_2 _ratio was measured in all patients within 24 hours after admission. In addition, the number of days on the ventilator and peak inspiratory pressure (PIP) rates were recorded, and presence or absence of ARDS was documented in accordance with the guidelines proposed by the American-European Consensus Conference on ARDS [13].

### Cytokine measurements

Blood was collected in serum separator collection tubes at the time of admission and 5 to 7 days thereafter (Figure 1). Blood was centrifuged at 1,320 rpm for 10 minutes, and serum was removed and then stored at -70°C until assayed. IL-1β, IL-2, IL-4, IL-5, IL-6, IL-7, IL-8, IL-10, IL-12p70, IL-13, IL-17, granulocyte colony-stimulating factor, granulocyte-macrophage colony-stimulating factor, interferon-γ, monocyte chemoattractant protein-1, macrophage inflammatory protein-1β, and TNF were measured using the Bio-Plex Human Cytokine 17-Plex panel in combination with the Bio-Plex Suspension Array System (Bio-Rad Laboratories Inc., Hercules, CA, USA). The assay was performed in accordance with the manufacturer's instructions. Briefly, serum samples were thawed, centrifuged at 4,500 rpm for 3 minutes at 4°C, and incubated with micro beads labeled with antibodies specific to one of the aforementioned cytokines for 30 minutes. After a wash step, the beads were incubated with the detection antibody cocktail, each bead specific to a single cytokine. After an additional wash step, the beads were incubated with streptavidin-phycoerythrin for 10 minutes, washed, and placed in the array reader for determination of the respective cytokine concentration.

### Statistical analysis

Unpaired Student's *t*-tests were used to compare differences in cytokine expression, differences in length of ventilation, PIP, and PaO_2_/FiO_2 _ratio between groups. Demographics were compared using *t*-tests or χ^2 ^tests. The Fisher's exact test was used to compare baseline variables. Data are expressed as percentages of means ± standard error of the mean, where appropriate. Statistical significance was accepted at a *P *value of less than 0.05. Statistics were run using SigmaStat 2004 (Systat Software Inc., Chicago, Illinois, USA). Multiple logistic regression was used to develop a prediction equation for determining the likelihood of mortality of burn patients with concomitant inhalation injury from early serum cytokine profiles. (To calculate a probability from the logistic equation shown in the Results section [below], transform the logit using Prob[YG9group] = 1/[1 + ExpBurned].) To assess the goodness-of-fit for the regression, the likelihood ratio test statistic and the mean, standard error of the mean, and Wald statistic for each parameter were examined.

## Results

Twenty-eight severely burned children with inhalation injury were studied. Patient demographics are shown in Table 1. Groups were of similar age, burn size, extent of third-degree burn, and time from burn to admission, but there were significantly more females than males in the survivor group.

**Table 1:** Patient demographics

Parameter	Survivors	Nonsurvivors
Number (*n*)	15	13
Age (years)	8 ± 2	9 ± 1
Sex (*n*; female/male)	3/12	9/4*
Burn to admittance (days)	3 ± 1	2 ± 1
TBSA (%)	65 ± 5	76 ± 4
Third degree (%)	53 ± 6	69 ± 5

Where applicable, data are presented as means ± standard deviation. *P < 0.05. TBSA, total body surface area.

**Figure 2:**
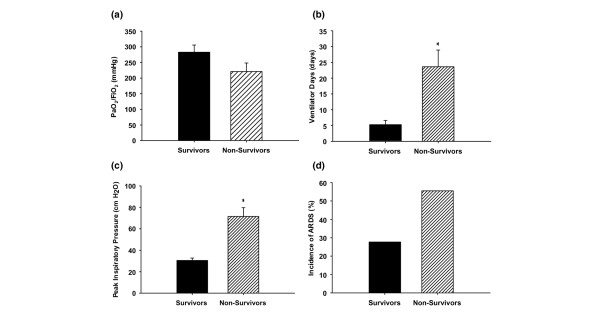
Nonsurviving pediatric patients with inhalation injury display more severe deterioration of lung function than their surviving counterparts. **(a) **The arterial oxygen tension (PaO_2_)/fraction of inspired oxygen (FiO_2_) ratio of severely burned children with inhalation injury who did not survive was lower than in those who survived (220 ± 27 mmHg versus 282 ± 23 mmHg). **(b) **Burn patients with inhalation injury who had a fatal outcome had significantly more ventilator days than children who survived (24 ± 5 days versus 5 ± 1 days). **(c) **Nonsurvivors exhibited significantly higher peak inspiratory pressure rates than survivors (71.5 ± 8.2 cmH_2_O versus 30.6 ± 2.1 cmH_2_O). **(d) **Severely burned children with concomitant inhalation injury who did not survive had a higher incidence of acute respiratory distress syndrome (ARDS) than did those who survived, which was not statistically significant (55.6% versus 27.7%). Bars represent means; error bars correspond to standard error of the mean. *P < 0.05.

Severely burned children with inhalation injury who did not survive exhibited lower PaO_2_/FiO_2 _rates within 24 hours after hospital admission when compared with children who survived (Figure 2a). Burn patients with inhalation injury who had a fatal outcome exhibited a significantly (*P *< 0.05) greater number of days on the ventilator than did children who survived (Figure 2b). Significantly higher PIP rates were observed in nonsurvivors than in survivors (*P *< 0.05; data shown in Figure 2c). Severely burned children with concomitant inhalation injury who did not survive had a higher incidence of ARDS as compared with those who survived, but this difference was not statistically significant (55.6% versus 27.7%).

Seventeen cytokine serum levels were significantly increased at the time of hospital admission, both in burned patients with inhalation injury who did not survive and in those who survived compared with levels in nonburned, normal pediatric patients (data not shown).

**Figure 3:**
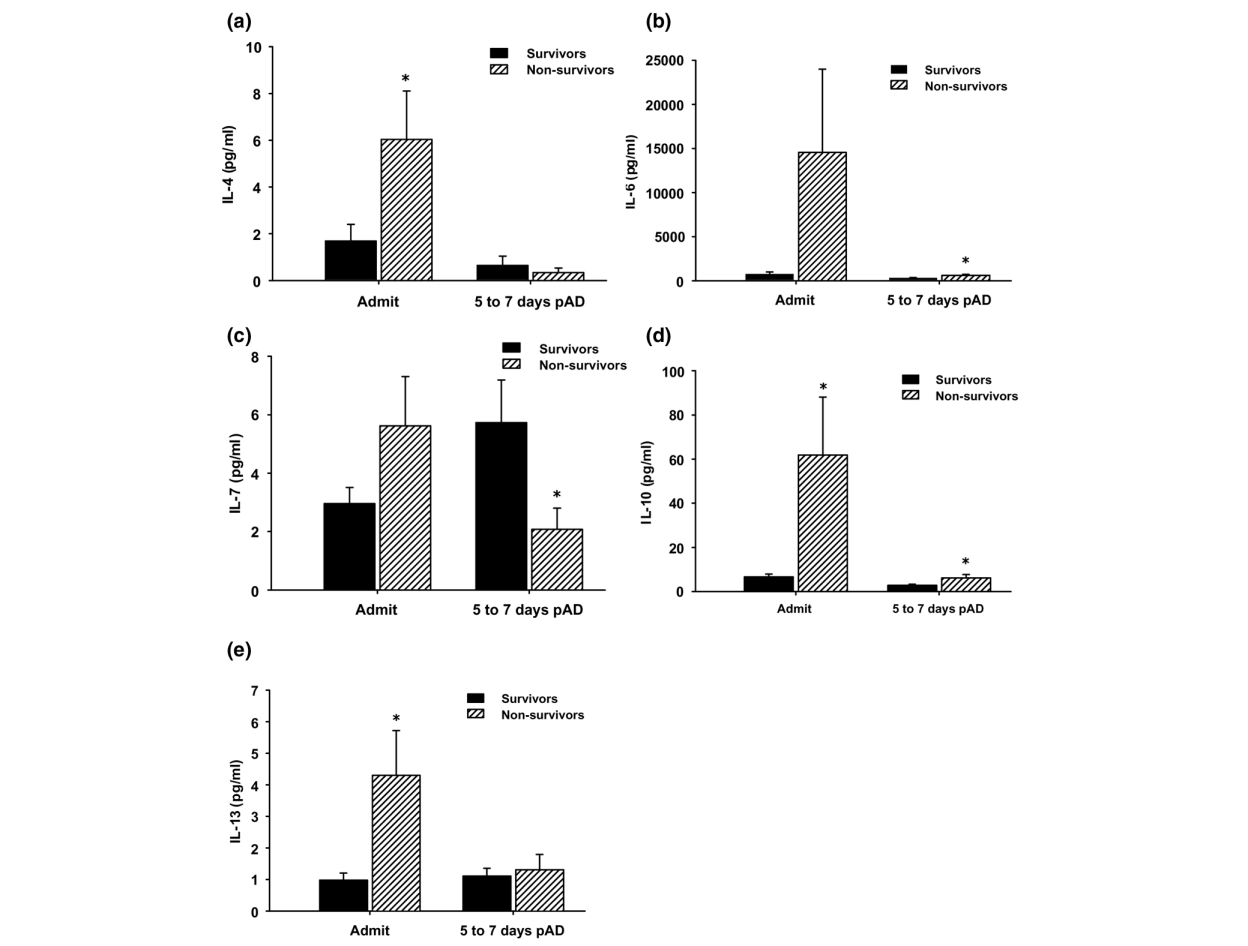
Cytokines are significantly altered in nonsurviving versus surviving patients who sustained inhalation injury. **(a) **IL-4 serum levels were significantly increased in the nonsurvivor group at admission compared with survivors (normal IL-4: 0 ± 0 pg/ml). **(b) **Nonsurvivors exhibited a significant increase in IL-6 serum levels 5 to 7 days after admission compared with the survivor group (normal IL-6: 8.7 ± 5 pg/ml). **(c) **Nonsurvivors exhibited a significant decrease in IL-7 serum levels 5 to 7 days after admission compared with the survivor group (normal IL-7: 3.8 ± 0.63 pg/ml). **(d) **IL-10 serum levels were significantly increased in the nonsurvivor group at admission and 5 to 7 days after admission compared with survivors (normal IL-10: 1.4 ± 0.3 pg/ml). **(e) **Nonsurvivors exhibited a significant increase in IL-13 serum levels upon hospital admission when compared with the survivor group (normal IL-13: 0.9 ± 0.2 pg/ml). Throughout the figure, histograms depict serum concentrations of the respective cytokine at steady state levels. Bars represent means; error bars correspond to standard error of the mean. *P < 0.05. pAD, post-admission days.

By comparing severely burned children suffering from inhalation injury who did not survive with those who survived, we found significant differences in serum levels of IL-4, IL-6, IL-7, IL-10, and IL-13 (Figure 3). Nonsurvivors exhibited a significant increase in IL-4 serum levels upon hospital admission when compared with the survivor group (*P *< 0.05; Figure 3a). IL-6 serum levels were significantly elevated in the nonsurvivor group at admission when compared with survivors (*P *< 0.05; Figure 3b). Nonsurvivors exhibited significantly lower IL-7 serum levels 5 to 7 days after admission compared with the survivor group (*P *< 0.05; Figure 3c). IL-10 serum levels were significantly increased in the nonsurvivor group at admission and 5 to 7 days after hospital admission compared with survivors (*P *< 0.05; Figure 3d). Nonsurvivors showed a significant increase in IL-13 serum levels at admission compared with the survivor group (*P *< 0.05; Figure 3e).

Serum levels of IL-1β, IL-2, IL-4, IL-5, IL-6, IL-7, IL-8, IL-10, IL-12p70, IL-13, IL-17, granulocyte colony-stimulating factor, granulocyte-macrophage colony-stimulating factor, interferon-γ, monocyte chemoattractant protein-1, macrophage inflammatory protein-1β, and TNF were not significantly different between the two groups.

We found a panel including IL-6, IL-7, and IL-10 to exhibit excellent predictive ability with respect to mortality (likelihood ratio test statistic: 7.1 [*P *< 0.008] and 8.5 [*P *< 0.01] at admission and 5 to 7 days after admission, respectively). The Wald statistic was significant for each of these three variables when this regression was run (*P *< 0.05). When any of the other 15 cytokines measured was added to the multiple logistic regression, the Wald statistic was not significant for the added cytokine. The other 15 cytokines were therefore not included in the multiple logistic regression analysis. A multiple logistic regression was conducted with the variables of mortality (dependent variable), IL-6, IL-7, and IL-10, and the following equations were obtained: Logit *P *= 1.551 - (0.343 × IL-10) and Logit *P *= 0.690 - (0.00662 × IL-6) + (0.869 × IL-7), at admission and 5 to 7 days after admission, respectively. The coefficients for IL-6 and IL-10 were negative, indicating that the risk for mortality increased as the levels of IL-6 and IL-10 increased. The coefficient for IL-7 was positive, indicating that the risk of mortality increased as the levels of IL-7 decreased.

The means, standard errors, and Wald statistics of the logistic regression coefficients are as follows: IL-10, 3.883 ± 0.174 (*P *= 0.049) upon admission; and IL-6, 4.570 ± 0.00289 (*P *= 0.022) and IL-7, 4.369 ± 0.416 (*P *= 0.037) 5 to 7 days after admission. When the other parameters were added sequentially, the following Wald statistics were obtained for the added variable: IL-4 (*P *= 0.196) and IL-13 (*P *= 0.158) at admission, and IL-10 (*P *= 0.143) 5 to 7 days after admission. Because some of the cytokines are highly correlated, the logistic regression is not improved by adding all five variables.

## Discussion

Smoke inhalation may lead to release of mediators that increase pulmonary artery pressure and cause secondary damage to the respiratory epithelium and the release of additional inflammatory molecules [3,8]. Lung injury resulting from smoke inhalation is associated with significant increases in the incidence of pneumonia and ARDS in thermally injured patients [14]. These may be exacerbated by early hemodynamic instability and massive burn edema, both of which are commonly observed in burn injury patients with smoke inhalation. Severe pulmonary dysfunction resulting from smoke inhalation therefore remains one of the leading contributors to mortality in patients with thermal injury [15]. Thus, early identification followed by appropriate management of those high-risk patients may lead to improved clinical outcome.

Alterations in inflammatory mediators, such as cytokines, are main contributors to the incidence of multiple organ failure and mortality in critically ill patients. Thus, in the present study we hypothesized that burned pediatric patients with inhalation injury who had a fatal outcome exhibited a different serum cytokine profile when compared with similar patients who survived. Patients divided into the two study groups were of similar age, burn size, extent of third-degree burn, and time from burn to admission. There were considerably more females than males in the survivor group. This does not constitute a concern, because we recently found that sex-specific differences in pediatric patients do not play a role in mortality rates [16].

Here we found that increases in inflammatory cytokines IL-4, IL-6, IL-7, IL-10, and IL-13 within the first 7 days after admission were strongly associated with the incidence of mortality in these patients. Patient mortality correlated with the incidence of ARDS, the number of ventilation days, the peak inspiratory pressure, and the PaO/FiO_2 _ratio in this population – parameters that are widely used to define inhalation injury.

Age, underlying disease, malnutrition, and infections have been studied as prospective clinical predictors of poor outcome after inhalation injury in addition to the PaO_2_/FiO_2 _ratio [6]. Despite its widespread use, the validity of the PaO_2_/FiO_2 _ratio as a tool for assessing pulmonary gas exchange has remained controversial [17]. González-Castro and colleagues [6] found a value of PaO_2_/FiO_2 _ratio above 100 mmHg 24 hours after admission to the intensive care unit to be associated with a lower mortality in patients who underwent lung transplantation. Yilmaz and coworkers [18] successfully utilized the PaO_2_/FiO_2 _ratio on day 3 after the onset of acute lung injury to assess hospital and 6-month mortality. In contrast, no correlation could be established between outcome in patients with severe lung injury and PaO_2_/FiO_2 _ratio by Krafft and colleagues [19]. Our data suggest that levels of PaO_2_/FiO_2 _below 220 mmHg at admission tend to be associated with fatal outcome in burn patients with inhalation injury. However, the higher values of PaO_2_/FiO ratio in nonsurvivors did not reach statistical significance when compared with those of patients who survived burn trauma with inhalation injury. This could be a result of the relatively small number of patients in our study. Pronounced deterioration in lung function in nonsurvivors was subsequently revealed by their significantly greater number of days on the ventilator and significantly higher PIP rates. An increased incidence of ARDS was also observed, but this was not statistically significant when compared with that in similar patients who survived the injury.

The aim of the present study was therefore to develop a means to predict the outcome of severely burned patients who sustained inhalation injury sufficiently early after admission to allow adequate measures to be implemented for their hospital management. Because of the relevance of inflammatory mediators as biomarkers in acute lung injury, we hypothesized that the levels of various cytokines were elevated early during the course of hospitalization in this patient population. Serum levels of IL-6, IL-8, and IL-10 have been evaluated in several clinical trials. Although not detectable in all patients at risk for developing ARDS [20-22], increased levels of IL-6 and the persistence of these levels have been strongly associated with mortality [20,23]. Similarly, in our study the increased levels of IL-6 determined during the first day after admission exhibited a strong correlation with outcome in patients who did not survive. There is mounting evidence that the immunomodulatory properties of IL-6 result in increased polymorphonuclear neutrophil (PMN)-mediated hyperinflammation, enhancing PMN cytotoxic potential and influencing host immunosuppression [11].

The proinflammatory cytokine IL-8 was also found to be increased in patients at risk for developing ARDS, although the association between plasma IL-8 levels and morbidity and mortality in small clinical studies has not been consistent [24-26]. In our study we found no correlation between elevated serum IL-8 levels and fatal outcome, even though burned patients with inhalation injury overall exhibited significantly increased serum levels of IL-8 when compared with healthy children. IL-8, mainly released from alveolar macrophages, is one of the most important contributors to the complex events that occur at reperfusion and is a key chemotactic factor for PMNs [27]. It dramatically enhances neutrophil transmigration through pulmonary endothelium and epithelium as well as PMN chemoattraction and activation [28]. However, because of its chemoattractant activity, it is likely that IL-8 contributes more to acute inflammation within the lung than in the circulation [25,26,29].

Unlike IL-8, we found IL-10 serum levels to be associated with fatal outcome. IL-10 has been demonstrated to inhibit alveolar macrophage production of proinflammatory mediators that are involved in severe lung injury. It has been also shown to play a role in downregulating HLA-DR expression on monocytes from septic patients and may play a role in modulating the host response to infections in these critically ill patients [30]. Schneider and coworkers [31] found that IL-10 is a critical mediator of immunosuppression after traumatic injury. Studies by Lyons and colleagues [32] indicated that increased IL-10 production correlates with subsequent septic events, and in the burn mouse IL-10 appears to induce decreased resistance to infection. Plasma IL-10 levels did not predict the development of ARDS in patients at risk but were found to be increased in patients with ARDS who did not survive [33]. IL-7 was found to have antiapoptotic effects on T cells via Bcl-2 expression, indicating that this cytokine plays an important role in supporting cell survival [34]. In a recent study by our group [1], this mediator was significantly decreased in pediatric burn patients with inhalation injury compared with similar patients without inhalation injury. In our study, decreases in IL-7 serum levels correlated with increased incidence of mortality in these patients. However, how this particular cytokine is modulated in response to inhalation injury is not known. In contrast, we found that the anti-inflammatory cytokines IL-4 and IL-13 were significantly increased upon admission. These cytokines are believed to be part of the underlying mechanisms for the development of ARDS [35]. IL-13 induces multiple features of allergic lung disease, including metaplasia and mucus hypersecretion, contributing to airway obstruction [36]. Increases in IL-13 mRNA in pulmonary tissue correlated closely with the incidence of ARDS in a rodent model [37]. An association with mortality was not found in these studies.

Compelling evidence that causally links elevation in various cytokine serum levels to poor patient outcome is lacking. However, it has been established that during the response following severe illness, release of various cytokines is not properly regulated [11,38]. Indeed, high blood levels of proinflammatory cytokines may lead to a debilitating condition known as autodestructive systemic inflammatory response syndrome [11]. In this condition both proinflammatory cytokines and anti-inflammatory cytokines appear in circulating blood, leading to septic shock, multiple organ dysfunction and immunosuppression, ultimately contributing to increased mortality [11,38].

## Conclusion

We believe that the results presented here from our relatively small patient cohort indicate that serum cytokine levels may be valuable outcome predictors in burn patients with inhalation injury, even though it is presently unclear whether their elevation arises from local pulmonary inflammation or an associated systemic inflammatory response. In contrast, despite its widespread use, the validity of the PaO_2_/FiO_2 _ratio as a tool for predicting mortality in patients with severe lung injury has remained controversial [6,16-18]. Determination of serum IL-6, IL-7, and IL-10 levels upon admission is convenient and simple, and may serve as an early indicator for identifying patients who have a greater risk for mortality after a burn with concomitant inhalation injury.

## Key messages

• Severely burned patients suffering from inhalation injury have a significantly increased risk for mortality compared with burned patients without inhalation injury.

• Alterations in inflammatory mediators, such as cytokines, are main contributors to the incidence of multiple organ failure and mortality in critically ill patients.

• Age, underlying disease, malnutrition, infections, and PaO_2_/FiO_2 _ratio are commonly utilized tools that may be used to predict poor outcome after inhalation injury.

• Alterations in IL-4, IL-6, IL-7, IL-10, and IL-13 appear to be associated with increased incidence of ARDS, number of days under ventilation, increased PIP, and lower PaO_2_/FiO_2 _ratio in this patient population.

• Early alterations in serum levels of IL-6, IL-7, and IL-10 may constitute useful predictive markers for identifying patients with high mortality after burns with concomitant inhalation injury.

## Abbreviations

ARDS = acute respiratory distress syndrome; FiO_2 _= fraction of inspired oxygen; IL = interleukin; PaO_2 _= arterial oxygen tension; PIP = peak inspiratory pressure; PMN = polymorphonuclear neutrophil; TNF = tumor necrosis factor.

## Competing interests

The authors declare that they have no competing interests.

## Authors' contributions

GGG gathered data, helped conducting the statistics, and wrote the manuscript. CCF performed experiments to obtain data, conducted the statistical analysis, and reviewed the manuscript. DNH gathered data, reviewed the analysis, and helped to write the manuscript. RPM helped to collect data and write the manuscript. MGJ designed the study, gathered data, conducted the statistical analyses, and reviewed the manuscript.
